# Bioengineering Case Study to Evaluate Complications of Adverse Anatomy of Aortic Root in Transcatheter Aortic Valve Replacement: Combining Biomechanical Modelling with CT Imaging

**DOI:** 10.3390/bioengineering7040121

**Published:** 2020-10-01

**Authors:** Cristiano Spadaccio, Laura Mazzocchi, Irina Timofeva, Laurent Macron, Carlo Nicola De Cecco, Simone Morganti, Ferdinando Auricchio, Francesco Nappi

**Affiliations:** 1 Department of Cardiothoracic Surgery, Golden Jubilee National Hospital, Clydebank G81 4DY, UK; cristianospadaccio@gmail.com; 2 Institute of Cardiovascular and Medical Sciences, University of Glasgow, Glasgow G12 8TA, UK; 3 Department of Civil Engineering and Architecture, University of Pavia, 27100 Pavia, Italy; laura.mazzocchi02@universitadipavia.it (L.M.); auricchio@unipv.it (F.A.); 4 Department of Imaging, Centre Cardiologique du Nord de Saint-Denis, 93200 St Denis, France; dr.irina.timofeeva@gmail.com (I.T.); laurentmacron@gmail.com (L.M.); 5 Division of Cardiothoracic Imaging, Nuclear Medicine and Molecular Imaging, Department of Radiology and Imaging Sciences, Emory University Hospital|Emory Healthcare, 1364 Clifton Road Northeast, Atlanta, GA 30322, USA; carlo.dececco@emory.edu; 6 Department of Electrical, Computer and Biomedical Engineering, University of Pavia, 27100 Pavia, Italy; simone.morganti@unipv.it; 7 Department of Cardiac Surgery, Centre Cardiologique du Nord de Saint-Denis, 93200 St. Denis, France

**Keywords:** transcatheter aortic valve replacement (TAVR), finite element analysis (FEA), biomechanical modelling, calcification, complications

## Abstract

Gated computed tomography (CT) might not adequately predict occurrence of post-implantation transcatheter aortic valve replacement (TAVR) complications in hostile aortic root as it would require a more complex integration of morphological, functional and hemodynamical parameters. We used a computational framework based on finite element analysis (FEA) to simulate patient-specific implantation. Application of biomechanical modelling using FEA to gated-CT was able to demonstrate the relation of the device with voluminous calcification, its consequent misalignment and a significant stent deformation. Use of FEA and other advanced computed predictive modelling techniques as an adjunct to CT scan could improve our understanding of TAVR, potentially predict complications and fate of the devices after implantation and inform patient-specific treatment.

## 1. Introduction

The use of transcatheter aortic valve replacement (TAVR) is still hampered by daunting complications mainly related to hostile anatomical characteristics of the aortic root. The presence of voluminous calcifications can severely impair device deployment leading to paravalvular regurgitation [1,2,3] valvular complications [4] or stress damage to the aorta [5]. Unfortunately, computed tomography (CT) imaging might not adequately predict the risk of these complications as it would require a more complex integration of morphological, functional and hemodynamical parameters. The use of finite element analysis (FEA) and other advanced computed predictive modelling techniques [4,5,6,7,8,9,10,11] in combination with CT scan could improve our understanding of TAVR, potentially predict complications and tailor patient-specific implantations.

## 2. Materials and Methods

We investigated the case of an 80 years-old woman undergone TAVR with a n° 26 CoreValve prosthesis and readmitted with acute coronary syndrome nine months after the index procedure. Biomechanical modelling and FEA was performed on pre-procedural CT scans to simulate implantation and infer potential complications deriving from the patient-specific aortic root anatomy and calcification distribution.

**Figure 1:**
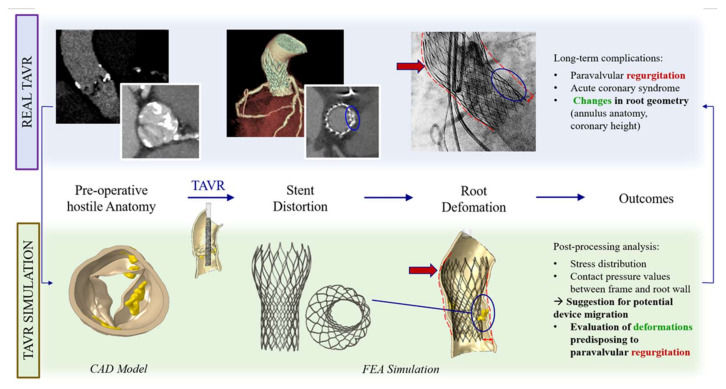
FEA may predict possible TAVR complications.

The computational framework adopted to simulate TAVR can be divided into four main phases. Step 1: processing of medical images; Step 2: construction of FEA-suitable models; Step 3: simulation of device implantation; Step 4: post-processing of the FE calculations (performed to extract quantitative measures of prosthetic stent deformation) and comparison with follow-up data. FEA was performed using the commercial FE solver Abaqus 6.14 by Dassault Systèmes (Simulia, Providence, RI, USA). The aortic root model was generated by considering a constant thickness of its wall at 2.5 × 10^−3^ m, while, for simplicity, native leaflets were reconstructed under the assumption of a uniform thickness of 0.5 × 10^−3^ m (Figure 1, Figures S1 and S2).

A linear isotropic elastic model was used to characterize leaflet tissues. Simplified St. Venant-Kirchhoff material properties were considered, with Young’s modulus E of 2 × 10^6^ Kg/m·s^2^ and a Poisson’s ratio ʋ of 0.45. The hyperelastic material model (proposed by Auricchio et al. [9]), described by a six-order reduced polynomial constitutive model represents the nearly incompressible nature of the cardiac root tissue. Density ρ was assumed equal to 1.1 × 10^3^ Kg/m^3^ for both aortic wall and valvular leaflets [4], while we assigned linear elastic properties to the calcified tissues adopting the following parameters: E = 10 × 10^6^ Kg/m·s^2^, ʋ = 0.35 and ρ = 2 × 10^3^ Kg/m^3^ [12]. Frictionless contact was assumed between the aortic root and the leaflets, while self-contact was applied to the leaflets. The interaction between calcifications and leaflets was defined by means of a kinematic coupling constraint technique, and a frictionless general contact was used to handle the interactions between calcifications and the aortic root inner surface. Material properties of the Nitinol alloy were used to model self-expandable CoreValve, according to constitutive laws reported in Auricchio et al. [13]. The entire TAVR procedure was computer-simulating starting with device crimping within its delivery system. The simplified catheter-defined as a cylindrical rigid surface- was then gradually removed with a sliding upwards movement to let the stent open, exploiting its superelastic behavior.

The subjects gave her informed consent for inclusion before they participated in the study. The study was conducted in accordance with the Declaration of Helsinki, and the protocol was approved by the Ethics Committee of 7/2020 (TAVR_CCN_2020_3).

## 3. Results

### 3.1. Clinical Outcomes

**Figure 2:**
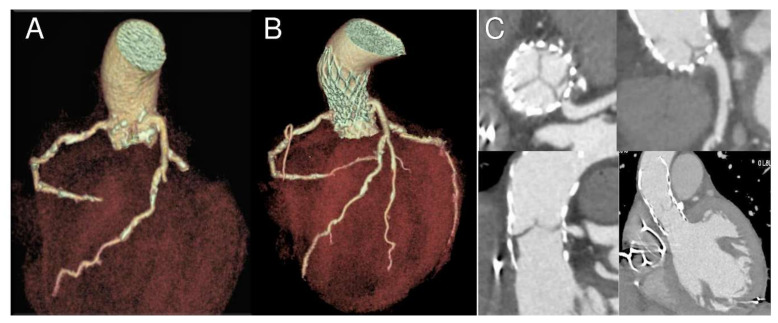
Nine-month follow-up gated CT scan demonstrating bioprosthesis malposition in respect to left main coronary. 3D reconstructions in longitudinal and cross-section before (**A**) and after the procedure (**B**,**C**). Please note coronary ostia proximity with calcifications and ostial stenosis due to partial obstruction by a portion of the THV frame (**C**).

**Figure 3:**
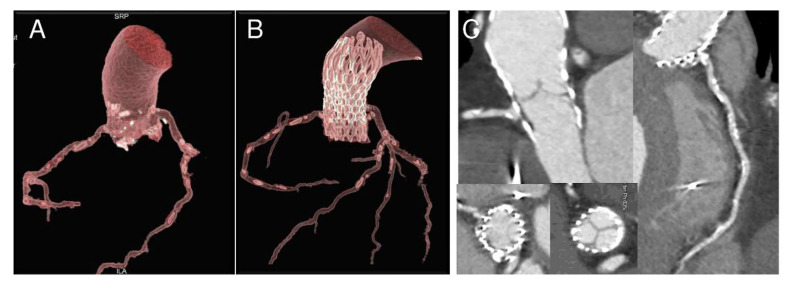
Nine-month follow-up gated CT scan demonstrating bioprosthesis malposition in respect to right coronary. 3D reconstructions in longitudinal and cross-section before (**A**) and after the procedure (**B**,**C**). Please note ostia proximity with calcifications and ostial stenosis due to partial obstruction by a portion of the bioprosthesis frame (**C**).

**Figure 4:**
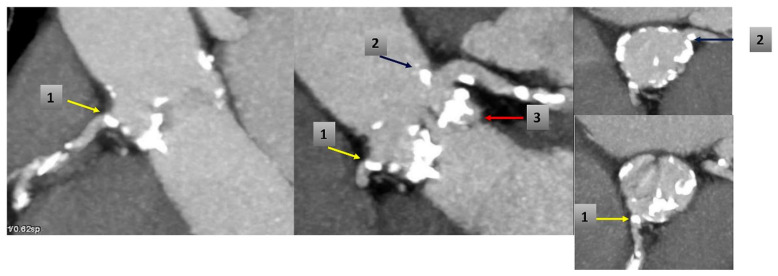
CT scan before TAVR shows: (1) RCA ostium calcification (yellow arrow); (2) calcification near ostium of the Left Main (blue arrow) and (3) calcified native valve under left coronary ostium (red arrow).

The index procedure was uncomplicated with no procedural atrioventricular block or embolic events and negligible aortic regurgitation. CT scan at the moment of readmission showed a marked change in the size of the annulus (preoperative calculated annular area 367 mm^2^, post-TAVR 272 mm^2^, change from baseline −95 mm^2^) and in the height of the coronaries relative to annulus (3 mm for the right coronary and 5 mm for the left coronary; change from baseline −7 mm and −6 mm respectively) (Table 1). Coronary ostia obstruction caused by pre-existing root calcifications was demonstrated and confirmed by angiogram, which showed an ostial stenosis of both the left main stem and the right coronary ostia with paravalvular leakage of the bioprosthesis (Figure 2, Figure 3 and Figure 4).

The patient underwent emergently to surgical aortic valve replacement and coronary artery bypass grafts on the left and right coronary system and made an unremarkable recovery being discharged on day 9.

### 3.2. Finite Element Analysis

**Figure 5:**
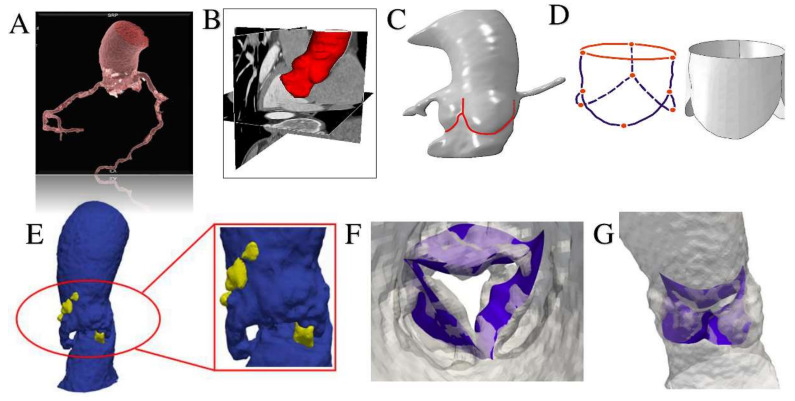
Using Rhinoceros 3D (McNeel & associates, Seattle, WA, USA) and Simulia Abaqus we performed an isogeometric analysis interfacing between computer aided design (CAD) and finite element analysis. (**A**,**B**) Pre-processing of aortic root model through the extraction of patient specific aortic valve geometry from medical CT images using VMTK (Vascular Modeling Toolkit, http://www.vmtk.org/) and MATLAB code (v.R2018b, Mathworks Inc-Natick, MA, USA). (**C**,**D**) Pre-processing of patient specific aortic valve. The modelling of leaflets was obtained using Rhinoceros 3D to Abaqus. (**E**) Generation of CAD model of the aortic wall (blue areas) with calcifications (yellow blocks). (**F**) Mapping of calcific leaflets from patient’s CT images. (**G**) Apposition of leaflets in patient’s aortic root model.

**Figure 6:**
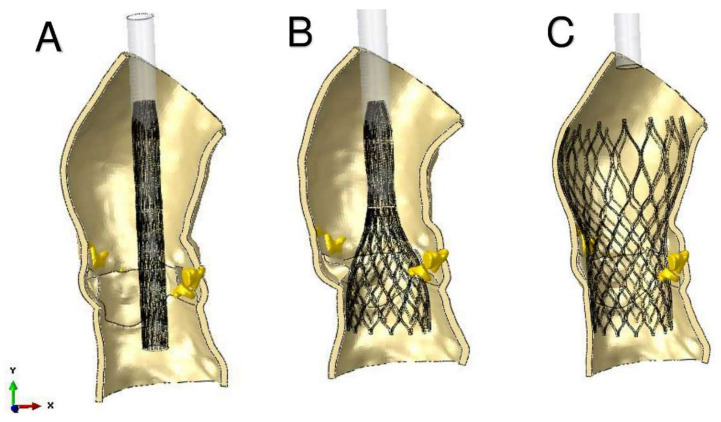
Main steps of simulation of TAVR using finite element analysis investigation. Catheter model has rigid material properties. (**A**) stent crimping; (**B**) catheter removing; (**C**) stent self-expansion.

**Figure 7:**
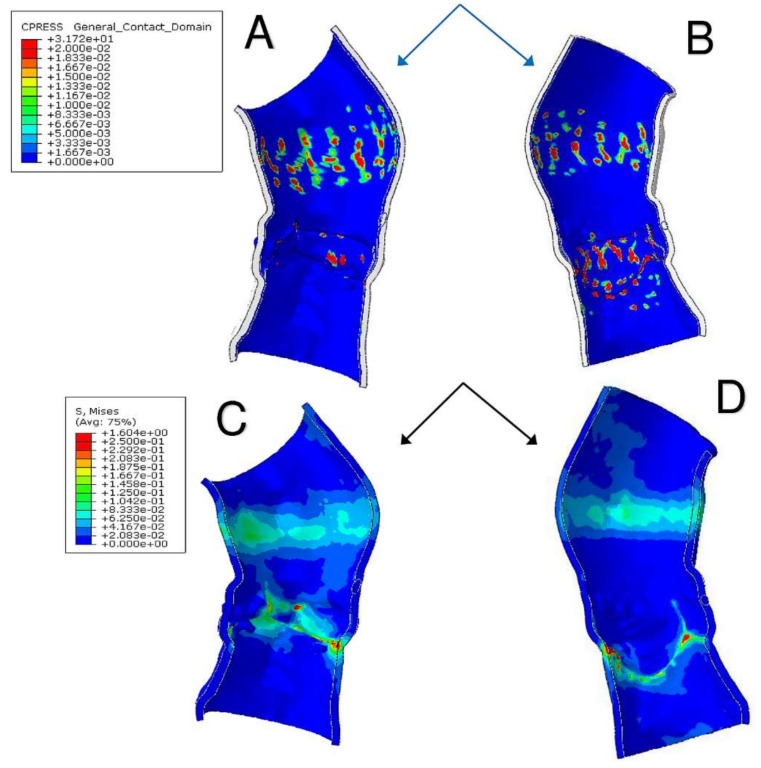
(**A**,**B**) Stent-root interaction area shows regions of aortic root elements with a contact pressure higher than zero. Quantitative measure of the device footprint onto the inner wall of the aortic root after expansion is shown. The evaluation of stent adhesion and anchoring to the aortic wall allows to reveal areas with inadequate stress and contact potentially leading to device migration. (**C**,**D**) Von Mises average stress measures the forces induced by the device expansion onto the inner wall of the aortic root and the quantitative and qualitative evaluation of their locations with higher peak stress. Higher values of stress also mean higher global anchoring force.

A precise geometrical model of the patient’s aorta, including the aortic root wall, native leaflets, and calcific plaques was developed from pre-operative CT images. On the basis of the recreated geometric model (Figure 5), we performed a FEA process, as previously described by Morganti et al. [6]. During the FEA simulation of TAVR procedure (Figure 6), Von Mises average stress distribution was computed to determine quantitative measure of the device footprint onto the inner wall of the aortic root after expansion. In Figure 7C,D stress peaks correspond to the sites with higher shear and higher global anchoring forces. As expected, greater values of stress are located in the contact areas between the stent frame and the patient-specific aortic wall (max = 1.604 × 10^6^ Kg/m·s^2^). In Figure 7A,B stent-root interaction area and contact pressure were measured. We found lower values of peak stress in correspondence of few scattered calcifications, suggesting for higher risk of device migration (max = 31.72 × 10^6^ Kg/m·s^2^).

**Figure 8:**
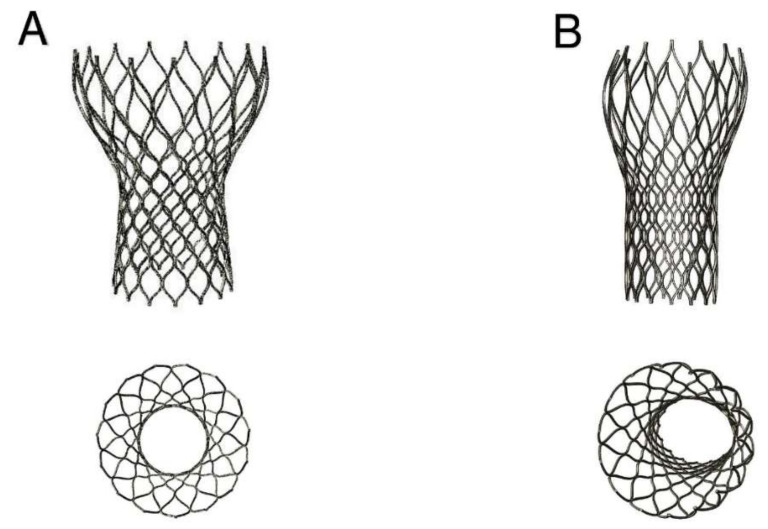
(**A**, top and bottom) Ideal CoreValve expansion with stent shape implantation; (**B**, top and bottom) Real deformation of the stent shape after implantation in the investigated patient’s root. Top: not complete expansion of the device; Bottom: asymmetric stent expansion.

**Figure 9:**
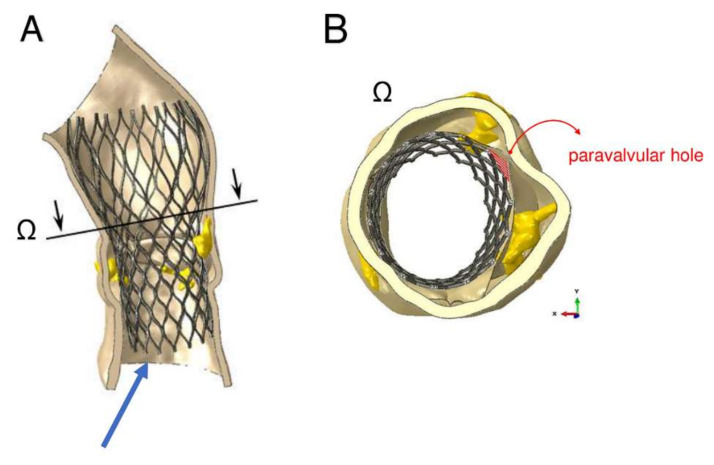
Areas of paravalvular leak can be measured as area of the orifices generated between the inner aortic wall and the device, after its expansion. The amount of blood flow regurgitation could be also quantitatively and indirectly evaluated from the dimension of paravalvular orifice. (**A**) cutting plane Ω correspondent to sino-tubular junction (STJ) of the model with incomplete expansion of the inferior part of the device (blue arrow). (**B**) Mismatch between expanded stent and inner aortic root wall is indicative of the development of paravalvular leakage (PVL) (red area).

The device was not properly aligned with the aortic root, thereby lacking complete basal attachment and showed stent deformation (Figure 8B). Figure 9A,B show how calcifications contributed to inadequate stent deployment and anomalous biomechanical behaviour of the device. Firstly, calcifications locally predisposed to paravalvular leaks. Secondly, a qualitative evaluation of the actual stent deformation clearly highlighted an incomplete and asymmetric expansion of the device (Figure 8B bottom and Figure 9A blue arrow). Over the nine months follow-up the continuous process of endothelialization and fibrotic encapsulation of such a deformed stent might have led to a general distortion of the entire root justifying the morphometric changes measured at post-implant CT scan. As shown by the cutting plane Ω in Figure 9, the geometrical distortion has also had the consequence of pushing the annulus cranially, putting in closer relationship the pre-existing calcifications with the coronary ostia.

Root calcification determined an asymmetric and incomplete expansion of the device with stent deformation. Distortion of the entire root geometry was noted as result of stent deformation and might have contributed to the approximation of coronary ostia to the pre-existing calcification.

## 4. Discussion

Despite remaining purely speculative, the observations made after biomechanical modelling and FEA in this case strengthened the idea that three-dimensional CT scan alone cannot predict the fate of TAVR device postimplantation. 

The proposed predictive biomechanical model can be a useful integration to the CT scan as was able to anticipate the potential incorrect deployment of the device, the presence of paravalvular leakage and the distortion of the stent due to voluminous calcifications. The geometric alterations and the location of calcifications, often close to the coronary ostia, can be at the root of daunting complications [4,5,6,7,8,9,10,11].

In this case we noticed dynamic changes of aortic anatomy many months after the procedure. These changes involved the diameter of the aortic anulus and the height of the coronary ostia, all significantly reduced in comparison to pre-TAVR. These findings are only in partial agreement with the ones by Madukauwa-David et al. [14], who performed a retrospective post-TAVR analysis of 4-D CT from 109 patients successfully implanted with Sapien 3 and CoreValve at a mean follow-up of 6.1 ± 7.0 months [15]. These authors did not find differences in aortic diameter post TAVR, while left coronary artery height decreased by 22% and right coronary artery height increased by 7.3% [14]. It is difficult to explain the reasons underlying these differences; however, it is possible to speculate that several anatomical and technical factors might have affected the implantation in our patient, which indeed was readmitted with a severe complication after an apparently successful TAVR. The CT scans analysed by Madukauwa-David et al. [14] belonged to patients implanted with no periprocedural problems and no further mid-term complications. Additionally, it should be considered that the localization, characteristics and distribution of the calcifications were not parameters included in their analysis. The nadirs of the native cusps may be displaced during implantation, leading to changes in the measured distances between the annulus and sino-tubular junction or coronary ostia. However, several other factors such as the definition of the annular plane in the preoperative planning, root elasticity, compliance, valve calcifications, etc., might have an effect. In our case we can hypothesize that the mentioned anatomical changes in association to the presence of residual calcifications had a role in the generation of the ischemic complication. The coronary ostia were “pushed” towards these pre-existing root calcifications as a consequence of root distortion. 

Authors acknowledge that these findings derive from the analysis of only one case, but, if confirmed by further investigations, the combined approach CT-FEA in the preoperative evaluation of TAVR candidates could really hold a promise to dramatically improve the management of these patients. Indeed, FEA could capture a significant amount of information potentially useful in predicting the fate of device implantation and inform patient-specific treatment. 

## 5. Conclusions

The use of FEA in the preoperative planning of TAVR permits not only the reconstruction of the patient-specific anatomy but also an in-silico simulation of the entire procedure in such specific anatomy. A combined FEA-CT approach could inform the physician on the potential risks or problems occurring after the implantation. In this case who developed long-term complications after TAVR, patient-specific preoperative FEA simulation of valve deployment anticipated that pre-existing root calcifications could have determined device misalignment and incomplete and asymmetric stent expansion. The subsequent device deformation predisposed to paravalvular regurgitation. The information provided by the combination of FEA with CT could assist and guide several aspects of the preprocedural planning such as the selection of the type of device (i.e., different profiles of the valves and type of stents), the best technique of implantation (transfemoral, direct aortic, transapical) or the use of strategies to protect or treat the coronary preventatively.

The routine use of finite element analysis as adjunct to CT scan could therefore provide complex integration of morphological and functional parameters predicting complications and informing patient-specific treatment.

## Figures and Tables

**Table 1:** Aortic root anatomy and measurements pre and post TAVR.

Procedural Gated Computed Tomography (CT). Aortic Root Anatomy Pre and Post TAVR		
Aortic Annulus (mm)	Before TAVR	After TAVR
Diameter max	26.6	19.5
Diameter min	17	17.7
Mean diameter	21.8	18.6
Perimeter	74	60
Area	367	272
Perimeter derived diameter	23.6	18.8
Area derived diameter	21.6	18.6
Ca score aortic valve	1073	-
Aortic annulus-RCA (height)	10	3
Aortic annulus-left main (height)	11	5
**Valsalva (mm)**		
Right cusp	24	24
Left cusp	26	26
NC	25	25
Perimeter	87	87
Area	541	541
**STJ (mm)**		
Diameter max	24	24
Diameter min	24	24
Mean diameter	24	24
Perimeter	75.4	75.4
Area (mm^2^)	452.2	452.2
**Ascending Aorta (mm)**		
Diameter max	29	29
Diameter min	28	28
Mean diameter	28.5	28.5
Perimeter	89.5	89.5
Area (mm^2^)	637.4	637.4

## Supplementary Materials

The following are available online at https://www.mdpi.com/2306-5354/7/4/121/s1, Figure S1. The patient presented with acute pulmonary oedema and severe aortic stenosis. She was in NYHA class 4 and had a Euroscore of 19.09. The transthoracic echocardiography showed a calculated aortic area of 0.74 cm^2^ and mean systolic gradient of 28 mmHg. Figure S2. Transthoracic echocardiography 3 week after anticoagulation shows complete normalisation of transvalvular gradient.
