# Supplementary material for: Bioengineering Case Study to Evaluate Complications of Adverse Anatomy of Aortic Root in Transcatheter Aortic Valve Replacement: Combining Biomechanical Modelling with CT Imaging
Source: Bioengineering (Basel). 2020 Oct 1;7(4):121. doi: 10.3390/bioengineering7040121 (PMC7712517; doi:10.3390/bioengineering7040121)
Supplement: Supplementary file 1 [file bioengineering-07-00121-s001.pdf]

bioengineering

Supplementary Materials

Bioengineering Case Study to Evaluate Complications of Adverse Anatomy of Aortic Root in Transcatheter Aortic Valve Replacement: Combining Biomechanical Modelling with CT Imaging

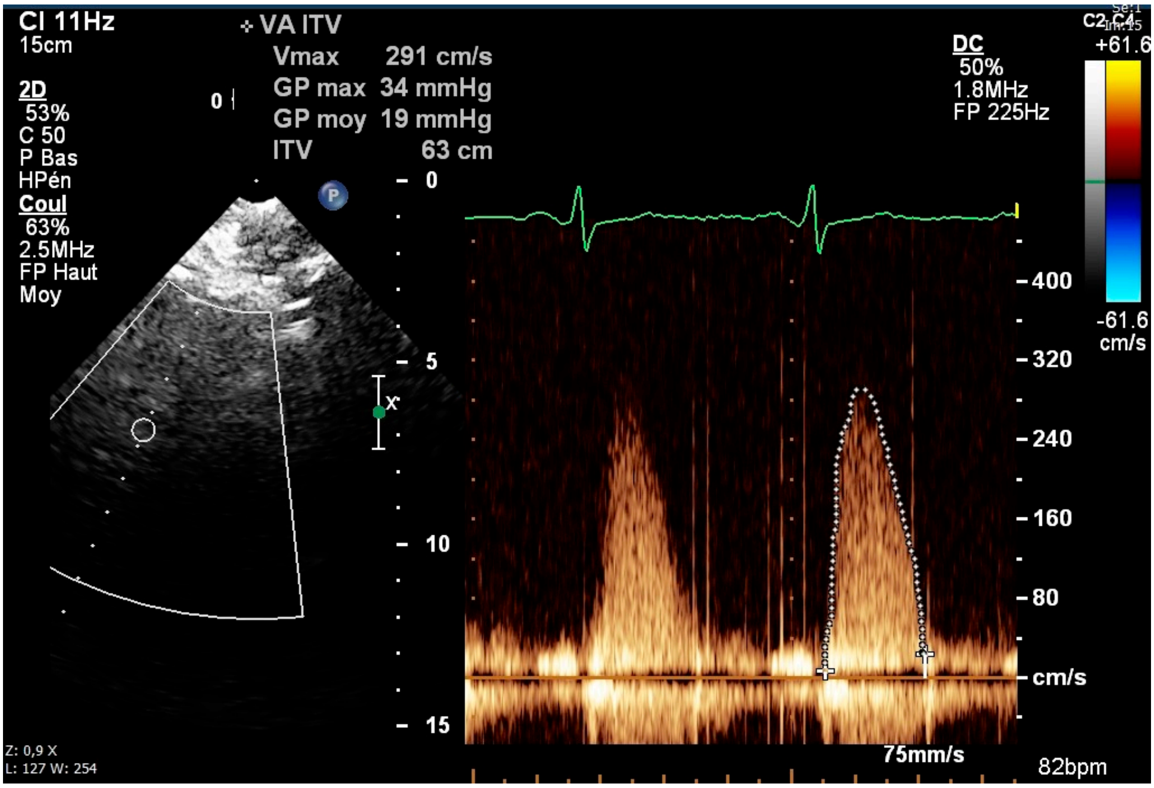

Figure S1. The patient presented with acute pulmonary oedema and severe aortic stenosis. She was in NYHA class 4 and had a Euroscore of 19.09. The transthoracic echocardiography showed a calculated aortic area of 0.74 cm² and mean systolic gradient of 28 mmHg.

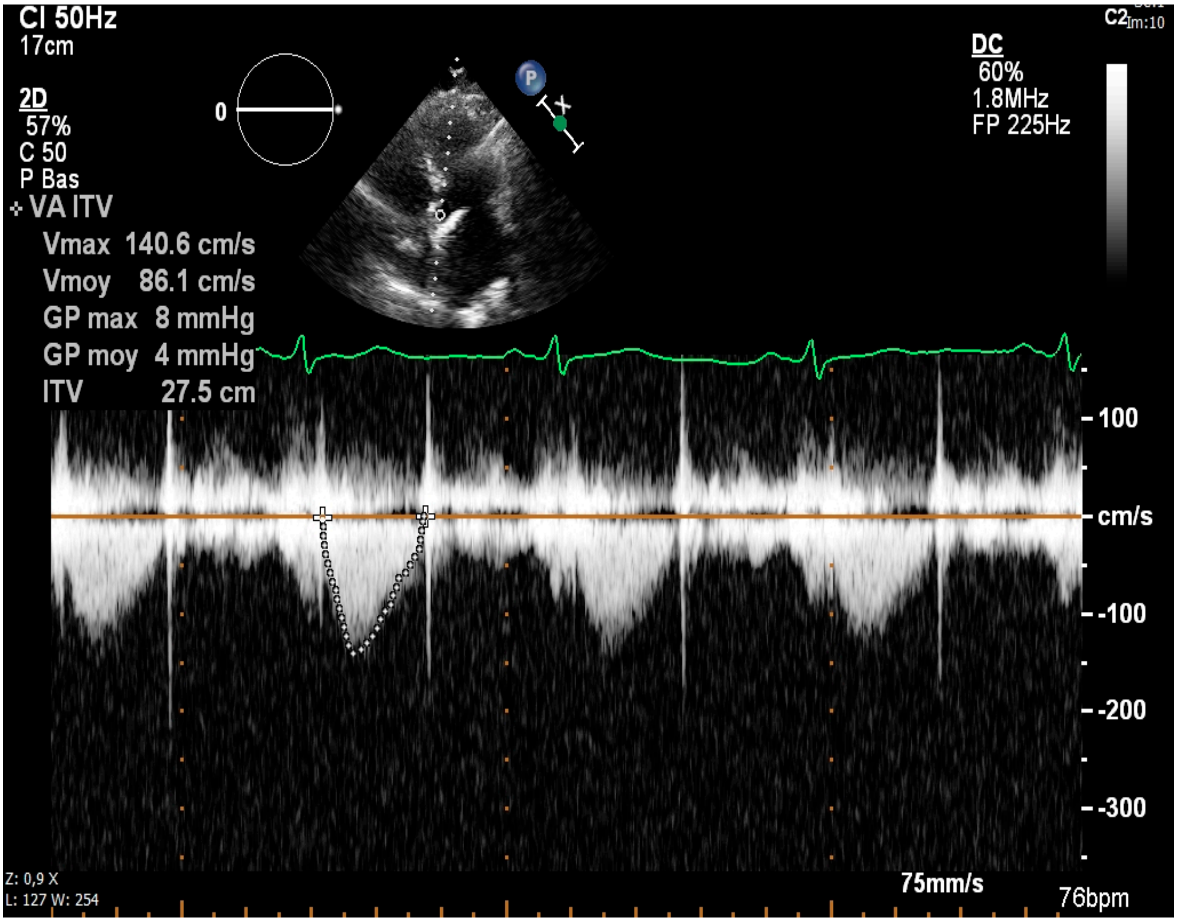

Figure S2. Transthoracic echocardiography 3 week after anticoagulation shows complete normalisation of transvalvular gradient.

Publisher's Note: MDPI stays neutral with regard to jurisdictional claims in published maps and institutional affiliations.

© 2020 by the authors. Submitted for possible open access publication under the terms and conditions of the Creative Commons Attribution (CC BY) license (http://creativecommons.org/licenses/by/4.0/).
